# Associations between Greenspaces and Individual Health: A Longitudinal Study in China

**DOI:** 10.3390/ijerph192013353

**Published:** 2022-10-16

**Authors:** Liping Liao, Minzhe Du

**Affiliations:** 1 School of Public Finance and Taxation, Guangdong University of Finance and Economics, Guangzhou 510320, China; 2 Center for the People’s Fiscal Development, Guangdong University of Finance and Economics, Guangzhou 510320, China; 3 School of Economics and Management, South China Normal University, Guangzhou 510006, China

**Keywords:** greenspaces, residents’ health, healthier habits, social cohesion, air quality

## Abstract

Using a longitudinal survey in China, this study identifies the effect of greenspaces on individual health in the aspects of self-rated health, mental health, feeling physical discomfort, and being hospitalized. The normalized difference vegetation index (NDVI) is used to measure the greenery cover of each city, and findings show that higher NDVI leads to the improvement of personal self-rated health status, and it also decreases the probability of being hospitalized, having symptoms of physical discomfort, and being lost in bad mood. The positive health effect of NDVI in the city is much more significant and larger for the middle-aged, the elderly, and the low-educated. The evidence of the three possible channels through which the NDVI of each city shows a positive correlation with individual physical and mental health is found. The increased NDVI in the city encourages residents to foster healthier habits (e.g., decreasing smoking or drinking, increasing sleeping hours), improves air quality and reduces air pollution in each city, and promotes the social cohesion of people, and so the health status of residents is enhanced. This study implies that increasing greenspaces in the city is an effective strategy to improve social welfare and residents’ health.

## 1. Introduction

As with the rapid economic development in China, the environmental protection and greening issues have attracted a lot of attentions. For example, the guidelines of the general office of the State Council on scientific greening were released in 2021, which emphasize the importance of removing illegal buildings in cities, increasing greenspaces, and raising the intensity of greening in megacities. According to data from the National Bureau of Statistics in China, urban green areas increased from 2240.29 to 3310.22 thousand hectares and the number of parks rose from 10,780 to 19,823, during the period from 2011 to 2020. On the other hand, although the mortality rate of people having chronic diseases has decreased in the last decade, the number of people having symptoms of chronic diseases has increased. Additionally, the overweight and unhealthy lifestyle problems of Chinese people are becoming more and more prominent. Therefore, how to improve the health status of residents in China remains an unsolved problem, and whether increasing greenspaces plays a positive role in the health status of residents should be further explored.

Recent studies regard greenspaces as one of the critical determinants of personal health or happiness and reach a consensus that the increased greenspaces or greenery cover show positive correlations with personal physical and mental health [1,2,3,4]. In the existing literature, less attention is paid to the empirical evidence on the effects of greenspaces in the context of developing countries, especially in China. Although some studies explore the role of greenspaces in personal health in China, they mainly focus on mental health or a single physical health indicator, such as self-rated health [5,6,7]. More subjective and objective health indicators concerning both physical and mental health are needed to analyze the link between greenspaces and people’s health status. Besides, the conclusions of the existing literature are based on cross-sectional data or a city sample, so one may worry that the results estimated with a small sample of a certain city cannot represent the general relationship between greenspaces and the individual health of the whole country. The empirical evidence of a longitudinal survey is needed to further enrich this line of literature. Lastly, the underlying mechanisms through which greenspaces have a beneficial effect on residents’ health should be examined. It is conducive to implementing a more targeted health policy and improving the social welfare of the whole society.

In this study, we match data from the China Family Panel Studies with the normalized difference vegetation index (NDVI) of each city to construct panel data when exploring the relationship between greenspaces and personal health. Findings show that if greenspaces increase, the probability of having mental problems or physical discomfort reduces, the self-rated health of residents improves, and the probability of being hospitalized decreases. In general, the average physical and mental health status is promoted when the greenery cover of each city increases. Besides, the health benefits are much larger for the elderly and the low-educated. The following three possible mechanisms are explored in this study. The rising greenspaces promote a positive change in residents’ healthy habits, and they tend to increase hours spent sleeping and physical exercise and decrease harmful health behaviors, including drinking or smoking, which is beneficial to residents’ health. Second, higher greenery cover in the city is conducive to the diffusion of air pollutants, which decreases the exposure to air pollutants for residents. Third, more outdoor activities and social interactions are encouraged when people live in a greener city, and they will feel a strong sense of belonging to the neighborhoods. Increased social cohesion is beneficial for the improvement of the physical and mental health of people.

Our study is different from the existing literature in the following ways. First, instead of using an indicator of mental health or physical health, we choose several measures to reflect the subjective and objective health of people more accurately, including self-accessed health, feeling physical discomfort, mental health, and hospitalization. Second, we construct panel data based on a nationally representative survey in China to explore the link between greenspaces and individuals’ health, which fills the gaps in empirical evidence of the effect of greenspaces with longitudinal data. Third, we examine the possible underlying mechanisms of the relationship between greenspaces and residents’ health, such as the improvement of air quality, the change in health behavior for people, and the increased social cohesion of residents.

Overall, we attempt to examine the following questions in this study:

(1) The effect of greenspaces on residents’ health status, such as self-rated health, doctor-diagnosed physical discomfort, mental health, and hospitalization;
(2) The heterogeneous effect of greenspaces on different groups categorized by education level, age, and gender;
(3) The potential channels through which greenery cover has a beneficial effect on residents’ health conditions, such as improving air quality, fostering healthier habits, or increasing social cohesion.

## 2. Literature Review

The multiple effects of greenspaces have been widely identified in both developed and developing countries, mainly in the aspects of individual health, academic performance, life satisfaction, quality of life, and so on [1,8,9]. For example, Stangierska et al. [8] examine the role of greenspace in the quality of life (QOL) for the urban population and conclude that satisfaction with greenery is a critical factor in determining their perceptions of whether the living neighborhood is an ideal place, while no direct relationship is found between the amount of greenspace and QOL. The amount of greenspace is related to urban infrastructure and affects residents’ QOL indirectly. Focusing on students’ academic performance, Browning and Rigolon [9] analyze the role of greenspace, concluding that mixed results are produced based on a small bundle of relevant studies, and further research is needed to enrich the evidence of mediation analysis. He et al. [1] emphasize the detrimental effect of a crowded neighborhood environment in high-density cities with structural equations and point out that eye-level greenspace can reduce the negative effect of urban density.

A consensus has been reached that higher exposure to greenspaces has a beneficial effect on individuals’ health both physically and mentally [2,3,4,10,11,12,13]. For instance, Klompmaker et al. [10] use the normalized difference vegetation index (NDVI) to explore the associations between greenspace exposure and the probability of being overweight for Dutch residents and find that outdoor physical activities can partly explain the above effect. Cole et al. [2] explore the health benefits of greenspace with data from New York City and find that greater exposure to greenspace is associated with better self-rated health, while it only shows a significant effect on people living in gentrifying neighborhoods. Glazer et al. [12] further provide evidence that there is a lower risk of preterm birth and underweight for people living in higher residential greenspaces. Fong et al. [4] draw a similar conclusion with the normalized difference vegetation index (NDVI) and emphasize the effect of greenspace varies across the different social-economic groups. Petraviciene et al. [3] focus on the underlying mechanism of the effect of greenspaces on children’s health and conclude that the distance to the park is one of the important mediators. From the perspective of mortality, Lee et al. [13] find that green vegetation can significantly reduce mortality for Asian people, while the underlying mechanism is not explored.

Apart from the empirical evidence of the health effects of greenspaces in developed or other Asian countries, some studies explore whether there is a link between greenspaces and residents’ health in the context of China [5,14,15]. Liao et al. [14] analyze the associations between greenspace and early childhood neurodevelopment and find that there is a significant increase in children’s PDI scores as the rise of greenspace exposure, especially for children of mothers whose BMI is lower than 24, and maternal physical activities and air pollution can act as possible mechanisms. Wang et al. [15] construct four measures of greenspace to access whether the natural environment improves people’s mental health with data from Guangzhou and find that the quality of eye-level greenspace is a critical factor in people’s mental health. Based on survey data of 26 neighbors in Guangzhou, Wang et al. [16] further conclude that greenspace quality plays an important role in narrowing the social-economic inequality of mental health. Qin et al. [5] point out that social cohesion is an important mediator of the relationship between greenspace and the mental health of people.

In general, previous studies mainly link greenspaces to people’s health in developed countries and from the perspective of mental health or physical health, such as self-rated health and overweight [2,10,11,12]. Although some studies explore the health benefits of greenspaces in the context of China, they mainly focus on people’s mental health and use small sample data from a certain city. Besides, a problem is that most of the existing literature explores the effects and the pathways based on cross-sectional data. Longitudinal studies revealing the potential influencing mechanisms behind the effect of greenspace on individual health are needed [17].

## 3. Data and Method

### 3.1. Data

This study uses the health status data from China Family Panel Studies (CFPS) between 2016 and 2018. It is a nationally representative survey in China, covering about 31 provinces and 125 cities (such as Xinyang, Zhoukou, Jinan, Shanghai, etc.) and including the whole area of the administration of cities. It provides personal and family information, such as education level, income, job history, family composition, and health status. It is a longitudinal survey administered by Institute of Social Science Survey, Peking University. Panel data were constructed during the period from 2016 to 2018 to examine the role of greenspaces in residents’ physical and mental health.

Three types of health status variables are selected from the survey. The first is residents’ physical health. The survey collects information about whether respondents felt any physical discomfort in the past two weeks. The answers range from 0 (no) to 1 (yes). It takes the value of 1 if residents suffer from some physical discomfort. Besides, self-rated health is usually used in empirical studies as one of the health measures [18]. We also use it as a health indicator and the answers to respondents’ self-rated health range from 1 (excellent) to 5 (poor). For convenience of analysis, we transform it from a negative indicator to a positive one and construct a dummy variable reflecting residents’ self-rated health. Higher value represents better self-rated health. The second type of health variables mainly measures residents’ mental health. The survey asks respondents several mental health questions, including “whether they felt lonely”, “whether they felt depressed”, “whether they felt that they cannot continue with their life” and “whether they felt sad” in the last week. The answers are scored from 1 (never) to 4 (most of the time). We use principal component analysis (PCA) to extract the first principal component as a mental health measure. Higher value represents that residents’ mental health is worse. The third type of health measure is actual health care use by residents. Respondents answered whether they were hospitalized in the last year. The above health variables includes both subjective and objective indicators and reflect residents’ health status more accurately.

The following overall greenery measure is used in this study to reflect the greenspaces of each city: normalized difference vegetation index (NDVI). It is one of the important parameters reflecting crop growth and nutrition information. The original vegetation data is obtained from MODIS (https://modis.gsfc.nasa.gov/ (accessed on 9 June 2022)). We first aggregated the monthly grid vegetation data into the annual one and then summarized it at the city level according to the average pixel values of each city to examine the role of greenery cover in residents’ health status. It is calculated based on the differences between the red and infrared bands following the following formula: NDVI = (infrared − red)/(infrared + red) [19]. The indicator value ranges from −1 to 1, and higher positive value represents more vegetation in the city. We mainly use NDVI in the empirical models to explore the effect of greenspaces on residents’ physical and mental health. Three types of variables are controlled in this study. The first is individual or family characteristics, including residents’ age, education level, income, marital status, hukou status, gender, and family size. The second type of controls are regional characteristics at the city level, such as per capita GDP (Gross Domestic Product), population density, the number of buses per 10,000 people, and the number of hospitals per 10,000 people. Besides, the weather data is collected from China National Meteorological Information Center, mainly including temperature and precipitation, and we aggregated it into the city level for each year.

We matched NDVI data with CFPS data and finally obtained a valid sample of 49,436 after excluding the missing variables. Table 1 provides the descriptive statistics of key variables.

### 3.2. Method

The fixed effect model is employed to explore the effect of greenspaces on residents’ physical and mental health. The baseline specification is as follows:

(1) $$Health_{ijt}=\alpha_{0}+\alpha_{1}NDVI_{jt-1}+\alpha_{2}X_{ijt}+\alpha_{3}W_{jt-1}+\alpha_{4}region_{jt-1}+\delta_{t}+\pi_{j}+\varepsilon_{ijt}\;$$

$Health_{ijt}$ is residents’ health status, including mental health, hospitalization, self-rated health, and whether they feel physical discomfort. The variable of our interest is $NDVI_{jt-1}$, representing the greenery level of each city at t−1. $X_{ijt}$ is a set of individual and family controls, such as age, marital status, family size, schooling year, gender, hukou status, and income. $W_{jt-1}$ reflects the weather condition at the city j at year t−1, including the annual temperature and precipitation at the city level. $region_{jt-1}$ are city characteristics, includes population density, the number of buses and hospitals per 10,000 people, and per capita GDP for city j at year t−1. $\delta_{t}$ is year fixed effect, and $\pi_{j}$ is city fixed effect. The standard errors are clustered at the city level. Considering hospitalization, self-rated health and physical discomfort are binary variables, we use Probit models to estimate the effect of greenspaces, while for mental health, the Ordinary least square method is used. In the robustness check, we substitute the health indicators and run the regressions again to verify the accuracy and reliability of estimated results.

## 4. Results

### 4.1. Baseline Results

The baseline results of the effect of NDVI on personal health status are shown in Table 2. Weather conditions, regional characteristics, city-fixed effects, and year-fixed effects are controlled in the models. In column (1), the effect of NDVI on personal self-rated health is reported. NDVI is significantly and positively correlated with residents’ self-accessed health, and more green vegetation can improve their self-rated health. It is significant at the 5% significance level. In columns (2)–(3), the link between greenspaces and residents’ health in the aspects of physical discomfort and mental health is explored. In general, higher greenspace exposure has a significant beneficial effect on residents’ mental health and physical discomfort. With the rise of NDVI, there is a lower probability for residents to feel physical discomfort and a higher probability for them to have a better mental condition. For example, as their greenspace exposure is higher, they are less likely to feel depressed, lonely, and sad. The results in Table 2 use the overall mental health index as the mental health measure with the principal component analysis. Besides, we also use an objective health indicator to examine the role of greenspaces in residents’ health, and the results in column (4) show that higher exposure to greenspaces will reduce residents’ probability of being hospitalized. It is significant at the 1% significance level. The above results are consistent with previous relevant literature that suggests urban greenery can raise people’s life satisfaction [1]. Therefore, results from Table 2 indicate that higher greenery cover has a significant positive effect on residents’ health, which improves their self-rated health and reduces the possibility of having a bad mood.

Table 2 also reports the results of controls on residents’ health (We also estimate the effect of NDVI on residents’ health without controlling for personal income, and the conclusion is consistent.). In columns (1)–(4), we find that the health status of the elderly is significantly poorer than that of the young. Their self-rated health is worse, they feel more physical discomfort, and they are more likely to feel depressed or lonely. Compared with females, males’ health status is much better. They are less likely to have been hospitalized in the last year and are more likely to rate their health status as good, which is significant at the 1% significance level. Family income is an important determinant in residents’ health. The high-income group is less likely to suffer from mental health problems and feel physical discomfort. Furthermore, per capita GDP is positively correlated with individual health. If people live in cities with higher economic development, their health status is better in the aspects of mental health and self-rated health. It may be attributed to more job chances, higher average income, and better public medical services in these cities.

### 4.2. Heterogeneous Results

#### 4.2.1. Heterogeneous Results of Greenspaces by Education Level

The link between people’s health status and education level is well recognized in the existing literature [20,21]. We further explore whether the effect of greenspaces varies across different educational groups. The high-educated group is defined as those who have obtained a high school education or above. Otherwise, residents are categorized into the low-educated group. The heterogeneous results are reported in Table 3. Panels A and B show that NDVI has a more beneficial effect on the low-educated group. More specifically, with the rise of greenery cover in each city, there is a lower probability for the low social-economic status group to feel physical discomfort and face mental problems. Besides, higher NDVI also reduces the probability of hospitalization for the low-educated group. It indicates that the exposure of low-educated residents to pollution and the surrounding environment may be higher, or that they are more likely to work outdoors. The improvement in greenspaces and the surrounding environment of each city may provide more health benefits for this group. On the contrary, the income of the high-educated or the high socio-economic group is usually relatively higher. They are less likely to face high-polluted or negative environments, or they may use avoidance behavior to avoid the risky effect of the surrounding environment [20]. The results in Table 3 show that NDVI is not significantly correlated with the physical and mental health status of the high-educated.

#### 4.2.2. Heterogeneous Results of Greenspaces by Age

The elderly are more likely to suffer from health problems and diseases [22,23]. Therefore, we further examine the role of greenspaces among different age groups. The sample is split into the following three groups: the young, the middle-aged, and the elderly. The young are defined as those who are aged 30 and below, and the elderly are those who are aged 45 and above. Otherwise, people are categorized as middle-aged. The results are reported in Table 4. Panels A, B, and C show that higher greenery cover in the city will improve residents’ health, especially the middle-aged or the elderly. NDVI is significantly and positively correlated with the middle-aged residents’ self-rated health, and the probability of feeling physical discomfort reduces with the rise of NDVI, which is significant at least at the 5% significance level. Besides, in panel C, columns (2)–(4) show that for the elderly group, more vegetation in the city plays a significant role in improving personal mental health and decreasing the probability of being hospitalized. These results are consistent with the previous health literature that suggests the elderly are sensitive to the surrounding environment and may suffer from more health problems when they live in fewer green environments [20]. Compared with the elderly, the health status of young people is usually better, and they are less likely to have diseases. Hence, the improvement of greenery cover shows less significant correlations with young people’s health.

#### 4.2.3. Heterogeneous Results of Greenspaces by Gender

We further examine how males and females respond differently to NDVI. Panels A and B of Table 5 show the results that an increase in the greenery cover of each city will mainly lead to an improvement in males’ health. In columns (1)–(3), compared with females, living in a more green environment decreases the probability of feeling physical discomfort for males, and they are less likely to feel depressed, lonely, or sad, which are significant at least at the 5% significance level. In column (4), NDVI plays an important role in the probability of being hospitalized for both males and females. In general, the positive change in greenery cover leads to a greater improvement in males’ health status. It is possible that males usually bear greater economic pressure, are more exposed to the surrounding environment, and acquire more health benefits from an improved environment. Men are more likely to ignore health problems and engage in risky health behaviors such as smoking or drinking. The increasing greenery cover is beneficial for men’s health in the aspect of physical discomfort or hospitalization [24].

### 4.3. Robustness Checks

#### 4.3.1. Changing Health Indicators

The results shown in the above sections are estimated with the following four health indicators: an overall mental health index, self-rated health, physical discomfort, and hospitalization. Although these subjective and objective health indicators can reflect individual health conditions comprehensively, it is still possible that some measurement errors are caused in the estimation process. To rule out such an interference, we select another health indicator in the survey to test whether the results are robust. In column (1) of Table 6, the results show that higher greenspaces significantly reduce the probability of seeing a doctor in the past two weeks for residents. It is consistent with our baseline results that the residents’ health can be improved by increasing the greenery cover of each city.

Besides, rather than using an overall mental health index, we further examine the correlation between NDVI with the following four mental health indicators in the survey to estimate the results again: whether residents feel sad, lonely, depressed, or that they cannot continue with their life. Four dummy variables are constructed according to the answers of the respondents. They take the value of one if residents answer “often” and “most of the time”; otherwise, these four health indicators equal zero. A higher value of these four indicators represents the worse mental health status of residents. The results are reported in columns (2) to (5) that as the rise of NDVI, residents are less likely to be lost in bad moods and feel sad or lonely. The above results indicate that even when we use each mental health indicator as a dependent variable in the models separately, the conclusion is credible and consistent.

#### 4.3.2. Using the Ordinary Least Square Method

The three health indicators (self-rated health, physical discomfort, and hospitalization) used in the baseline models are dummy variables, and we use Probit models to estimate the effect of greenspaces on residents’ health. In this section, we employ the ordinary least square method to estimate the regressions again and test whether the effect of greenery cover is robust. The results are reported in Table 7. After changing the estimation method, the conclusion remains consistent. The increase in NDVI is positively correlated with residents’ self-rated health, which is significant at the 1% significance level. Besides, the increased NDVI has a beneficial effect on residents’ health in the aspect of feeling discomfort or being hospitalized. These results are similar to the baseline results, and a consistent conclusion can be drawn that more greenery in the environment improves residents’ health status measured with subjective and objective indicators.

## 5. Potential Mechanisms

In the above sections, the associations between greenspaces and residents’ physical and mental health are explored, and greenery cover is identified as an important factor in improving residents’ health status. In this section, the underlying mechanisms through which greenspaces show significant correlations with residents’ health are further explored. To sum up, three mechanisms are analyzed. First, healthier habits are fostered for residents when the green cover in the city increases, and residents tend to increase their hours spent on physical exercise and sleeping and decrease the frequency of risky health behaviors (smoking or drinking). Second, as a sink to purify air pollution and accelerate pollutant diffusion, more greenspaces can improve air quality and enhance residents’ health. Third, residents’ social interactions are strengthened through social activities, such as square dancing or outdoor walking, and the relationship with neighbors becomes much better after the green cover of the city increases, which contributes to the improvement of residents’ health.

### 5.1. Fostering Healthier Habits

People will increase the frequency of walking or physical activities, which may bring health benefits to them, and physical exercise has a great effect on residents’ well-being and health [25]. Therefore, when residents are exposed to a greener environment, they may increase the hours spent on physical exercise to keep fit and improve their health [26]. Besides, a greener environment can help residents release their pressures and make them feel more comfortable [5]. Therefore, a greener environment may change residents’ lifestyles or help them foster healthier habits, such as decreasing the frequency of smoking or drinking and increasing sleeping hours.

To test the above hypothesis, four lifestyle indicators were chosen from the survey. The first indicator is smoking behavior. Respondents are asked whether they smoked in the past month. A dummy variable is constructed based on their answers. It equals one if residents smoked. The second indicator is the frequency of drinking. A dummy variable is constructed, and it takes the value of one when people answer that they drink alcohol at least three times a week. The third indicator is residents’ physical exercise time in the last week. It is a continuous variable. The last indicator is residents’ sleeping hours.

The results are shown in Table 8. In column (1), the more greenery environment of each city is negatively correlated with their smoking behavior, and the probability of smoking for residents is reduced with the rise of NDVI. Similarly, the results in column (2) show that higher vegetation cover in the city can decrease the frequency of drinking for residents, which is significant at the 5% significance level. Besides, columns (3) to (4) show the results of how the green environment affects residents’ time use in physical exercise and sleeping. Living in a greener city will significantly raise residents’ physical exercise time, which is significant at the 1% significance level. It is consistent with the existing literature that increased physical activities are caused because of the improvement of a greener environment. Higher exposure to a greener environment also has a beneficial effect on reducing pressures and increasing their sleeping hours. Overall, higher greenery cover in the city can improve residents’ health through changing residents’ lifestyles and helping them foster healthier habits, such as decreasing the frequency of risky health behaviors, increasing their sleeping hours, and increasing their physical exercise hours.

### 5.2. Improving Air Quality

Increased greenery cover in each city is an effective measure for improving air quality, and it can raise the speed of pollutant diffusion [27,28,29]. The negative impact of air pollution is well recognized. It increases the probability of having respiratory diseases or chronic diseases and decreases residents’ happiness and life satisfaction [20,30]. Therefore, residents living in a city with more greenery cover may face a less negative effect of air pollution, which has a beneficial effect on their physical and mental health status.

To test the above hypothesis, the following three indicators were selected from the Chinese Ministry of Environmental Protection (CEMP): the concentrations of PM2.5, PM10, and SO_2_ (μg/m^3^). PM2.5 and PM10 represent the particulate matter with diameters smaller than 2.5 or 10 μm, respectively. SO_2_, called sulfur dioxide, is the most common sulfur oxide and one of the most important air pollutants. The above indicators are widely used in the air pollution literature to reflect the air quality and pollutions condition of each city [31,32]. We follow the existing literature, aggregate the above indicators to the city level for each year, match CFPS data with air pollution data, and use them as air pollution measures. All three indicators are continuous variables. The results are reported in Table 9. In column (1), the higher greenery cover of each city reduces the PM2.5 concentration, which is significant at the 1% significance level. The results of columns (2)–(3) support the similar conclusion that the concentrations of PM10 and SO_2_ have decreased because of the rise of greenery cover in the city. It indicates that with the rise of greenery cover, more air pollutants are absorbed, and the air quality improves significantly. It verifies our hypothesis that a greener environment plays a critical role in decreasing air pollution and improving air quality, which is beneficial for personal physical and mental health status.

### 5.3. Increased Social Cohesion

Higher greenery cover in the city will encourage people to join in outdoor activities, such as walking outside or “square dancing,” which can promote social interaction, help residents make friends, and raise their satisfaction with the living environment [5,33]. Social cohesion brings a sense of belonging for residents, raises their happiness, and has a beneficial effect on residents’ health [34]. Therefore, social cohesion may act as a mediator between greenery spaces and residents’ physical and mental health.

To test the above mechanism, three indicators of community life were selected from the survey to measure the extent of residents’ social cohesion. The first is residents’ feelings about the public facilities around their community, such as education, medical, and transportation. The second question asked residents how the relationship between neighbors in their community is. The third indicator is whether they are emotionally attached to the community. Answers to the above questions range from one (very good) to five (very poor). We use the PCA method to extract the first principal component as a measure of community life. It is a negative indicator that a higher value represents a lower social cohesion of residents. The results are shown in column (4) of Table 9. Higher greenery cover of each city significantly enhances residents’ social cohesion, improves their social interaction in the community, and raises their satisfaction with the neighborhoods, which is helpful for their physical and mental health. The above results support the hypothesis of the third mechanism that social cohesion can act as a possible channel through which greenery cover improves personal health status.

## 6. Discussion

The existing literature has explored a similar topic, and greenspaces have been regarded as one of the determinants of personal well-being or health status. However, most studies focus on the physical health or mental health of people [7,35], or they mainly analyze the role of greenspace in children’s health [14,36]. The associations between greenery cover and individuals’ mental and physical health need to be further examined, which is the main content of our study. Compared with the results of existing literature, apart from the mental health effect of greenspaces (e.g., Zhang et al. [7]), we also confirmed that higher greenery cover has a positive effect on residents’ physical health. Although Balseviciene et al. [36] mainly explore the effect of greenspace with children sample, our study indicates that the conclusion of the beneficial effect of greenspace is applicable to both adults and children.

Most of the studies do not dig out the possible underlying mechanism of the effect of greenery cover [7,35,36]. Considering higher greenery exposure releases personal pressures and encourages residents to take part in physical exercise, the change of healthy habits is used as one of the mechanisms. Second, as a sink to absorb and diffuse the air pollutants, the greenery cover of each city is highly correlated with local air quality, and the improved air quality can partly explain the above effect. Third, the existing literature about the health effects of greenery cover does not pay attention to the mediating role of social cohesion. More outdoor activities and social interactions are encouraged when the greenery cover increases, which finally has a beneficial effect on individuals’ health. It sheds light on the importance of social cohesion or social trust when the role of greenery cover in personal outcomes are examined. These mechanisms are different from the existing studies and further explain why the health status of some residents is higher from the perspective of greenery cover.

The results of the correlation between greenery cover and residents’ health are possibly biased if residents have changed their living address and lived elsewhere. When people change their residence, the improvement in residents’ health may be erroneously attributed to the effect of the greenery cover of another city, leading to over-estimation or under-estimation problems. Or, people may be unaccustomed to the new residence, so they have a lower health performance, such as having a bad mood in the new neighborhood. Therefore, we also estimate the regression models after deleting the sample who have changed living addresses. Findings show that even when we use a sample who do not change their residence, the results are consistent. It means that the effect of greenery cover on personal health is robust, and the empirical evidence in our study is credible.

There are also some limitations in the following aspects. First, although we focus on the effect of a greenery environment on residents’ physical health and mental health with both subjective and objective indicators (including self-rated health or having physical discomfort), more general and accurate conclusions can be drawn if more doctor-diagnosed health indicators are used to measure mental health, such as whether residents take antidepressants. Second, due to data limitations, NDVI is used to measure the greenery cover of each city. More detailed results will be provided if we know the distance to the parks for residents or the area of the nearest park, namely, the distribution of greenspaces across the city. Third, other mechanisms may also mediate the effect of greenery cover on personal health status.

Future research can further examine the relationship between greenery cover and residents’ health if medical records data of patients is provided, such as whether residents take medicines. More abundant conclusions and baseline results are obtained when more personal medical information is collected in a large-scale survey. Second, the relationship between greenery cover at the neighborhood level and residents’ health can be further explored. It shows the differentiated health responses of residents to the surrounding environment when residents live in a greener neighborhood or near a large park. Third, more mechanisms can be explored to explain the indirect effect of greenery cover, such as the family atmosphere. More greenery cover may increase the frequency of family activities, such as family outings, which provide health benefits to residents. Besides, facing the shock of COVID-19, residents may pay more attention to outdoor activities, such as walking, running, or square dancing. Further research can focus on the interaction effect of COVID-19 and the greenery cover of each city on residents’ health status. It is possible that the outbreak of COVID-19 strengthens the positive effect of the greenery cover on residents’ health since some residents have experienced self-quarantined at home.

## 7. Conclusions and Implications

Our study is one of the first studies to explore the link between greenery cover in the city and residents’ health status in China, based on a nationally representative survey and longitudinal data. The following four subjective and objective health indicators are selected in this paper: self-rated health, mental health, feeling physical discomfort, and being hospitalized. The rise of greenery cover in the city significantly enhances residents’ health status, and it has a larger effect on the middle-aged and the elderly, the low-educated, and males. Three possible mechanisms are confirmed in this study. Higher vegetation cover is beneficial for residents’ health through promoting positive changes in health habits for residents (such as increasing sleeping hours and decreasing the frequency of smoking and drinking) or strengthening social cohesion. Besides, improved air quality also acts as one of the underlying mechanisms of the effect of greenery cover on personal health.

Several policy implications can be inferred from the empirical evidence in this study. As an effective tool to raise the health status of residents, the greenery cover of each city should be improved continuously. Considering the heterogeneous effect of greenspaces, the health benefits of the whole society can be achieved to a greater extent, if the vegetation cover is mainly improved in communities where the low-educated or the elderly are concentrated. As a social welfare to residents, increasing greenery cover not only releases residents’ pressures directly but is also beneficial for residents’ health through reducing air pollution.

## Figures and Tables

**Table 1:** Summary statistics of key variables.

Variable	Definition	Mean	Std.Dev.
***Panel A. Dependent variable***			
Self-rated health	How would you rate your health status. Binary variable, 0 = poor, 1 = good.	0.671	0.470
Hospitalization	Were you hospitalized due to illness in the last year? Binary variable, 0 = no, 1 = yes.	0.124	0.330
Physical discomfort	Did you feel physical discomfort during the past two weeks? Binary variable, 0 = no, 1 = yes.	0.305	0.461
Sadness	Did you feel sad last week? 1(*never*)-4(*most of the time*).	1.499	0.702
Continuing with life	Did you feel that you could not continue with your life last week? 1(*never*)-4(*most of the time*).	1.210	0.552
Loneliness	Did you feel lonely last week? 1(*never*)-4(*most of the time*).	1.454	0.743
Depression	Did you feel depressed last week? 1(*never*)-4(*most of the time*).	1.700	0.770
***Panel B. Greenery measure***			
NDVI	Normalized difference vegetation index at the city level.	0.296	0.108
***Panel C. Individual and family characteristics***			
Marital status	Binary variable, 1 = married, 0 = otherwise.	0.780	0.414
Age	16–60.	47.550	17.180
*Hukou*	Binary variable, 1 = rural *hukou*, 0 = otherwise.	0.733	0.442
Family size	The number of family members.	4.289	2.046
Schooling year	The schooling years of respondents.	6.821	4.929
Gender	Binary variable, 1 = male, 0 = female.	0.496	0.500
Income	The logarithm of family income (CNY).	10.840	1.087
***Panel D. Weather controls and regional characteristics***			
Temperature	The annual temperature at the city level (centigrade).	163.100	185.900
Precipitation	The annual precipitation at the city level (millimeter).	1297	814.200
Population density	Population density at the city level (10,000 people/ square kilometer).	0.056	0.052
Bus	The number of buses per 10,000 people at the city level.	3.791	4.272
Per capita GDP	Per capita GDP at the city level (CNY/people).	10.270	1.112
Hospital	The number of hospitals per 10,000 people at the city level.	0.411	0.385

**Table 2:** Effect of greenspaces on individual health.

	(1)	(2)	(3)	(4)
	Self-Rated Health	Physical Discomfort	Mental Health	Hospitalization
NDVI	0.004 **	−0.211 **	−0.143 **	−0.232 ***
	(0.002)	(0.103)	(0.058)	(0.076)
Marital status	−0.174 ***	−0.038 **	−0.350 ***	−0.015
	(0.015)	(0.019)	(0.019)	(0.027)
Age	−0.022 ***	0.013 ***	0.004 ***	0.022 ***
	(0.002)	(0.001)	(0.000)	(0.001)
*Hukou*	0.017	−0.051 *	0.057 ***	−0.074 ***
	(0.014)	(0.028)	(0.018)	(0.028)
Family size	−0.013 ***	−0.011 **	−0.015 ***	−0.005
	(0.001)	(0.004)	(0.004)	(0.005)
Schooling year	0.101 ***	−0.055 ***	−0.079 ***	−0.020 **
	(0.012)	(0.011)	(0.006)	(0.009)
Gender	0.217 ***	−0.282 ***	−0.311 ***	−0.088 ***
	(0.013)	(0.017)	(0.013)	(0.023)
Income	0.087 ***	−0.041 ***	−0.113 ***	−0.005
	(0.007)	(0.011)	(0.008)	(0.010)
Population density	−20.952 ***	38.000 *	21.576 ***	16.024
	(1.465)	(22.537)	(8.212)	(13.825)
Per capita GDP	0.083 ***	−0.114 **	−0.087 ***	−0.076 *
	(0.001)	(0.049)	(0.026)	(0.046)
Bus	−0.021 ***	0.031	0.027 **	−0.013
	(0.000)	(0.020)	(0.012)	(0.011)
Hospital	0.001	0.149 ***	0.075 *	0.006
	(0.003)	(0.052)	(0.039)	(0.043)
Weather conditions	YES	YES	YES	YES
City-fixed effect	YES	YES	YES	YES
Year-fixed effect	YES	YES	YES	YES
*N*	49,436	49,436	49,436	49,436

Notes: Standard errors in parentheses, cluster at the city level. ***/**/* indicates significance at the 1%/5%/10% levels. Weather controls include the annual mean of temperature and the annual mean of precipitation at the city level. Probit models are used in columns (1), (2), and (4) to examine the relationship between greenspaces and resident’s health. The ordinary least square method is used in column (3). Coefficients in the table reflect the effect of each independent variable on personal health.

**Table 3:** Heterogeneous effect of greenspaces by education level.

	(1)	(2)	(3)	(4)
	Self-Rated Health	Physical Discomfort	Mental Health	Hospitalization
***Panel A. The low-educated group***				
NDVI	0.006	−0.259 **	−0.166 **	−0.223 ***
	(0.004)	(0.126)	(0.072)	(0.084)
*N*	37,524	37,524	37,524	37,524
***Panel B. The high-educated group***				
NDVI	0.000 (0.028)	−0.078 (0.108)	−0.040 (0.092)	−0.228 (0.178)
*N*	11,912	11,912	11,912	11,912
Individual and family characteristics	YES	YES	YES	YES
Weather conditions	YES	YES	YES	YES
City-fixed effect	YES	YES	YES	YES
Year-fixed effect	YES	YES	YES	YES

Notes: Standard errors in parentheses, cluster at the city level. ***/** indicates significance at the 1%/5%/10% levels. Weather controls include the annual mean of temperature and the annual mean of precipitation at the city level. Individual and family characteristics include residents’ age, hukou status, family size, marital status, gender, and income. Panel A shows the effect of NDVI on the health conditions of low-educated residents, while panel B reports the relationship between NDVI and high-educated residents’ health status.

**Table 4:** Heterogeneous effect of greenspaces by age.

	(1)	(2)	(3)	(4)
	Self-Rated Health	Physical Discomfort	Mental Health	Hospitalization
***Panel A. The young***				
NDVI	−0.183	0.216	−0.149	−0.300 *
	(0.177)	(0.145)	(0.117)	(0.157)
*N*	9701	9701	9701	9701
***Panel B. The middle-aged***				
NDVI	0.121 *** (0.019)	−0.272 ** (0.119)	0.063 (0.112)	−0.219 (0.165)
*N*	11,355	11,355	11,355	11,355
***Panel C. The elderly***				
NDVI	−0.039	−0.288 **	−0.208 ***	−0.239 **
	(0.070)	(0.133)	(0.080)	(0.098)
*N*	28,380	28,380	28,380	28,380
Individual and family characteristics	YES	YES	YES	YES
Weather conditions	YES	YES	YES	YES
City-fixed effect	YES	YES	YES	YES
Year-fixed effect	YES	YES	YES	YES

Notes: Standard errors in parentheses, cluster at the city level. ***/**/* indicates significance at the 1%/5%/10% levels. Weather controls include the annual mean of temperature and the annual mean of precipitation at the city level. Individual and family characteristics include residents’ schooling years, hukou status, family size, marital status, gender, and income. Panels A, B, and C report the results of the effect of NDVI on the health status of the young, the middle-aged, and the elderly, respectively.

**Table 5:** Heterogeneous effect of greenspaces by gender.

	(1)	(2)	(3)	(4)
	Self-Rated Health	Physical Discomfort	Mental Health	Hospitalization
***Panel A. Females***				
NDVI	−0.046	−0.115	−0.130	−0.204 **
	(0.059)	(0.132)	(0.086)	(0.101)
*N*	24,907	24,907	24,907	24,907
***Panel B. Males***				
NDVI	0.071 (0.081)	−0.309 *** (0.096)	−0.153 ** (0.077)	−0.261 *** (0.087)
*N*	24,529	24,529	24,529	24,529
Individual and family characteristics	YES	YES	YES	YES
Weather conditions	YES	YES	YES	YES
City-fixed effect	YES	YES	YES	YES
Year-fixed effect	YES	YES	YES	YES

Notes: Standard errors in parentheses, cluster at the city level. ***/** indicates significance at the 1%/5%/10% levels. Weather controls include the annual mean of temperature and the annual mean of precipitation at the city level. Individual and family characteristics include residents’ schooling years, hukou status, family size, marital status, age, and income. Panels A and B provide the results of the associations between NDVI and the health status of females and males, respectively.

**Table 6:** Changing health indicators.

	(1)	(2)	(3)	(4)	(5)
	Seeing a Doctor	Sadness	Continuing with Life	Loneliness	Depression
NDVI	−0.278 **	−0.140 ***	−0.277 ***	−0.177 ***	−0.127 ***
	(0.112)	(0.001)	(0.034)	(0.001)	(0.013)
Individual and family characteristics	YES	YES	YES	YES	YES
Weather conditions	YES	YES	YES	YES	YES
City-fixed effect	YES	YES	YES	YES	YES
Year-fixed effect	YES	YES	YES	YES	YES
*N*	49,436	49,436	49,436	49,436	49,436

Notes: Standard errors in parentheses, cluster at the city level. ***/** indicates significance at the 1%/5%/10% levels. Weather controls include the annual mean of temperature and the annual mean of precipitation at the city level. Individual and family characteristics include residents’ schooling year, hukou status, family size, marital status, age, gender, and income. Column (1) shows the results of substituting health indicator with seeing a doctor. Columns (2)–(5) reports the effect of NDVI on each mental health indicator, rather than measuring personal mental health with an overall mental health index.

**Table 7:** Employing the ordinary least square method.

	(1)	(2)	(3)
	Self-Rated Health	Physical Discomfort	Hospitalization
NDVI	0.005 ***	−0.071 **	−0.045 ***
	(0.000)	(0.035)	(0.016)
Individual and family characteristics	YES	YES	YES
Weather conditions	YES	YES	YES
City fixed effect	YES	YES	YES
Year fixed effect	YES	YES	YES
*N*	49,436	49,436	49,436
adj. *R*^2^	0.144	0.073	0.060

Notes: Standard errors in parentheses, cluster at the city level. ***/** indicates significance at the 1%/5%/10% levels. Weather controls include the annual mean of temperature and the annual mean of precipitation at the city level. Individual and family characteristics include residents’ schooling year, hukou status, family size, marital status, age, gender, and income. The ordinary least square method is used in this table as a robustness check.

**Table 8:** Fostering healthy habits.

	(1)	(2)	(3)	(4)
	Smoking	The Frequency of Drinking	Hours Spent in the Physical Exercise	Sleeping Hours
NDVI	−0.024 ***	−0.112 **	1.083 ***	0.061 ***
	(0.003)	(0.057)	(0.011)	(0.000)
Individual and family characteristics	YES	YES	YES	YES
Weather conditions	YES	YES	YES	YES
City fixed effect	YES	YES	YES	YES
Year fixed effect	YES	YES	YES	YES
*N*	49,436	49,436	49,436	49,436

Notes: Standard errors in parentheses, cluster at the city level. ***/** indicates significance at the 1%/5%/10% levels. Weather controls include the annual mean of temperature and the annual mean of precipitation at the city level. Individual and family characteristics include residents’ schooling year, hukou status, family size, marital status, age, gender, and income. Columns (1)–(4) shows the correlations between NDVI and health habits of residents.

**Table 9:** Improving air quality.

	(1)	(2)	(3)	(4)
	PM2.5	PM10	SO_2_	Social Cohesion
NDVI	−3.197 ***	−1.791 ***	−3.012 ***	−1.113 **
	(0.161)	(0.140)	(0.165)	(0.535)
Individual and family characteristics	YES	YES	YES	YES
Weather conditions	YES	YES	YES	YES
City-fixed effect	YES	YES	YES	YES
Year-fixed effect	YES	YES	YES	YES
*N*	49,436	49,436	49,436	24,974

Notes: Standard errors in parentheses, cluster at the city level. ***/** indicates significance at the 1%/5%/10% levels. Weather controls include the annual mean of temperature and the annual mean of precipitation at the city level. Individual and family characteristics include residents’ schooling year, hukou status, family size, marital status, age, gender, and income. The questions about community life are only asked in the 2016 survey, and so there are sample differences in column (4). The independent variable is multiplied by 10,000 in Table 9. Columns (1) to (3) shows the relationship between NDVI and air pollutant concentrations, and column (4) reports the effect of NDVI on social cohesion level of residents.

## Data Availability

The data presented in this study are openly available in China Family Panel Studies (CFPS) and the International Scientific & Technical Data Mirror Site.
